# Topological photonic crystal fiber

**DOI:** 10.1126/sciadv.ady1476

**Published:** 2025-11-12

**Authors:** Bofeng Zhu, Kevin Hean, Stephan Wong, Yuxi Wang, Rimi Banerjee, Haoran Xue, Qiang Wang, Alexander Cerjan, Qi Jie Wang, Wonkeun Chang, Yidong Chong

**Affiliations:** ^1^ School of Physical and Mathematical Sciences, Nanyang Technological University, Singapore 637371, Singapore.; ^2^ Centre for Disruptive Photonic Technologies, Nanyang Technological University, Singapore 637371, Singapore.; ^3^ School of Electrical and Electronic Engineering, Nanyang Technological University, Singapore 639798, Singapore.; ^4^ Center for Integrated Nanotechnologies, Sandia National Laboratories, Albuquerque, NM 87185, USA.; ^5^ Department of Physics, The Chinese University of Hong Kong, Shatin, Hong Kong SAR, China.; ^6^ School of Physics, Nanjing University, Nanjing 210093, China.

## Abstract

Photonic crystal fibers (PCFs) provide a versatile platform for various applications, thanks to the flexibility with which light guiding can be customized by modifying the fiber geometry. We realize a PCF with guided modes produced by photonic band structure topology rather than conventional mode-trapping mechanisms. The design, which is compatible with the stack-and-draw fabrication process, consists of a cross-sectional photonic topological crystalline insulator with a disclination. A bulk-defect correspondence produces degenerate topological modes, lying below the cladding light line. We use various theoretical methods to confirm their topological origins, including a spectral localizer that makes minimal assumptions about the band structure. Our experiments on the fabricated fiber show it transmitting visible to near-infrared light with low losses of 10 to 20 decibels per kilometer, which do not increase substantially when the fiber is bent. A comparable solid-core PCF of conventional design exhibits substantially higher bending losses. Optical fibers based on topological modes hold promise for improved performance and versatile functionalities.

## Teaser

A photonic crystal fiber is demonstrated using topological protection from its nontrivial band structure.

## INTRODUCTION

Photonic crystal fibers (PCFs) (*1*–*9*) are a subset of the broader class of photonic crystals: structures that use wavelength-scale modulations to manipulate light (*1*, *10*). Although photonic crystals have been used in high-performance lasers (*11*) and solar cells (*12*), arguably their most important applications are in PCFs, including high-power light delivery (*5*), supercontinuum light generation (*13*), and sensing (*14*). In recent years, a new approach to designing photonic crystals, called topological photonics, has emerged (*15*–*17*). This involves engineering photonic band structures similar to those found in topological phases of condensed matter, thereby giving rise to distinctive photonic modes that owe their existence to topological “correspondence principles” rather than conventional light-trapping mechanisms (*18*–*21*). Aside from providing avenues for fundamental research into band topology, topological photonics holds promise for device applications due to the robustness of topological modes against certain forms of disorder. There is a substantial amount of ongoing research on topological waveguides (*22*, *23*) and resonators (*24*–*26*), mostly based on the photonic crystal slab geometry, which is well suited to photonic band structure engineering due to the availability of powerful fabrication techniques like photolithography. Bringing topological photonics into PCFs, however, has proven more challenging.

Previous theoretical proposals (*27*–*31*) for topological photonic crystal fibers (TPCFs) have been hampered by incompatibility with existing fiber fabrication methods, including the lack of mechanical stability in preform stacking, reliance on delicate structural features or precise index modulation in glass, and other issues. Many other designs for implementing topological photonics in PhC slabs are difficult to adapt to PCFs for similar reasons. Recently, researchers have developed a multicore PCF whose cores are placed in a Su-Schrieffer-Heeger configuration, a one-dimensional (1D) lattice with topological end-states (*32*), but that design is based on the arrangement of the waveguiding cores, not the topological properties of the underlying PCF band structure.

Here, we design and experimentally implement a TPCF that guides light via robust topological modes based on defect states in topological crystalline insulators (TCIs). Although studies of topological band structures usually focus on boundary states created by the bulk-boundary correspondence principle (*15*), lattice defects such as disclinations (*33*) can also host localized topological states due to the related bulk-defect correspondence principle (*34*), as shown in recent experiments (*20*, *21*, *35*, *36*). [Photonic defect modes based on other topological principles have also been demonstrated (*37*–*40*).] In particular, TCIs—structures with nontrivial band topology sustained by lattice symmetries (*17*–*19*)—can host topological disclination states associated with fractionalized spectral charge, as shown recently in a pair of photonics-based experiments (*20*, *21*). Taking a similar approach, we design a TPCF whose 2D cross section is a photonic TCI with a central disclination. The overall structure, consisting of glass capillaries and rods of different radii, is compatible with the standard stack-and-draw method for fabricating PCFs. The TCI hosts disclination states localized around a central air hole; when extruded along the fiber’s *z* axis, these form a set of 10 waveguide modes that we call guided topological defect modes (GTDMs). To show that the GTDMs originate from nontrivial photonic band topology, we first establish that the underlying bulk TCI is topologically nontrivial and that the disclination traps fractional charges (*20*, *21*); then, we use a recently developed “spectral localizer” framework (*41*–*47*) to specifically identify the GTDMs as topological states.

These GTDMs have an unexpected feature that is advantageous for waveguiding: Despite originating from topological gaps in the bulk band structure, they do not reside in those gaps nor in the bulk bands. Instead, they lie below the lowest bulk band, i.e., below the cladding light line. This property, which is not disallowed by theoretical principles (*18*, *19*), serves to inhibit cross-talk with the bulk states in a manner analogous to solid-core PCFs (*6*). It allows for robust, broad-band waveguiding without a complete 3D bandgap of the sort used in earlier demonstrations of guided defect modes (which used designs that are hard to implement in a PCF) (*36*, *48*, *49*). We experimentally measure the TPCF’s transmission loss to be 10 to 20 dB/km across much of the visible to near-infrared range. When the TPCF is strongly bent (two loops of radius 1 cm), the measured output power decreases by less than 5 dBm throughout the operating frequency range, whereas a conventional solid-core PCF fabricated with the same equipment and facilities experiences a decrease of up to 25 dBm. The GTDMs also have an interesting structure that can be exploited for spatial and/or polarization multiplexing. In the future, it will be interesting to see how such TPCFs, after appropriate performance optimizations, compare to state-of-the-art PCFs based on conventional design principles. Our work also points to optical fibers as an important platform for future work on topological photonics, with many unexplored application possibilities.

## RESULTS

### Fiber design and implementation

**Fig. 1.**
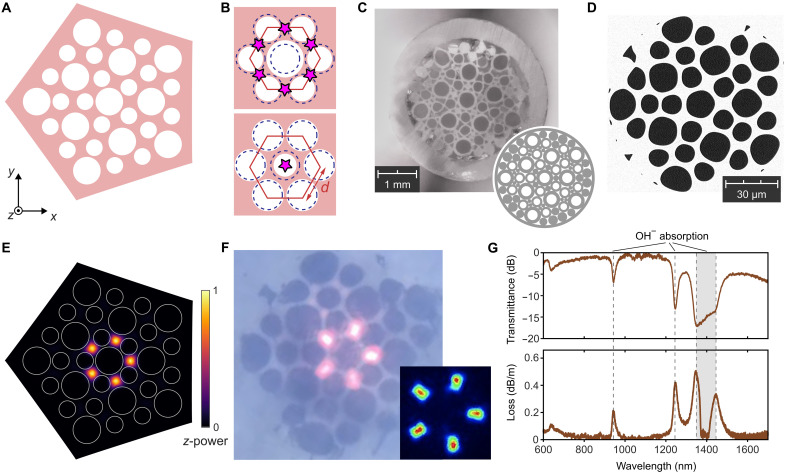
TPCF implementation. (**A**) Schematic of the cross-sectional photonic structure, comprising a TCI with a disclination. (**B**) Unit cell (red lines) for the disclination-free TCI, which has a $C_{6v}$ symmetry. Starting from a triangular lattice of equal air holes (dashed circles), the hole radii are alternately increased and decreased. For two different choices of modulation, the Wannier centers (magenta stars) are located at the unit cell’s sides (top plot) or center (bottom plot). (**C**) Photograph of the drawn fiber cane. Inset: Stacking arrangement of the preform. (**D**) Scanning electron microscope image of the TPCF’s end face. (**E**) Calculated intensity profile (power flow in the *z* direction) for a disclination state of the structure from (A) at $k_{z}d/2\pi =2$ . The air holes are indicated by white circles. (**F**) Optical microscope photograph of a 100-m TPCF, with a supercontinuum light source at the opposite end. Inset: Infrared camera image of the same. (**G**) Measured transmittance (top plot) for a 67-m TPCF and transmission loss (bottom plot) using the same source.

The TPCF is translationally invariant along the fiber axis (denoted by *z*), with a pattern of air holes in the transverse (*x*-*y*) plane (Fig. 1A). This cross-sectional pattern forms a 2D photonic crystal with a lattice defect (*33*, *34*). Specifically, it is a photonic TCI of the Wu-Hu type (*22*, *30*, *50*), containing a disclination that hosts topological disclination states (*20*, *21*, *34*) to be used for waveguiding.

In the absence of the disclination, the cross-sectional structure has a $C_{6v}$ point group symmetry, with each unit cell containing two air holes (top plot of Fig. 1B). We introduce a disclination of Frank angle −π/3 into the lattice using a “cut-and-glue” process (*19*, *51*) (see text S1 and fig. S1). This yields the fivefold rotationally symmetric structure of Fig. 1A, in which the hole radii are $R_{1}=0.57d$ and $R_{2}=0.35d$ , where *d* is the nearest-neighbor center-to-center distance in the original periodic lattice (see Fig. 1B).

By design, such a structure is compatible with the stack-and-draw process (*2*, *3*) for fabricating PCFs. The air holes are formed from glass capillaries of two different radii, stacked within a glass jacket with major gaps filled by additional solid glass rods, as shown in the inset of Fig. 1C. This stacking arrangement is derived from the configuration of Fig. 1A by a jam-packing procedure. The preform is drawn into a fiber cane (Fig. 1C), which is then further drawn into a fiber with a diameter of 310 μm. A scanning electron microscope image of the TPCF’s end face is shown in Fig. 1D, showing that the drawing process has filled in most of the interstitial air holes while also slightly deforming the main air holes. Further details about the fabrication procedure are given in text S1.

The key operating principle for the TPCF is that disclinations in TCIs can bind fractional spectral charges, giving rise to localized disclination states (*20*, *21*, *34*). Such states are tied to the TCI topology via a bulk-defect correspondence, similar to the bulk-boundary correspondence governing topological corner states in disclination-free TCIs (*18*, *19*) (in some circumstances, disclination states can even act as probes for band topological features that boundary probes cannot pick up). Whereas previous experimental realizations of topological disclination states (*20*, *21*) have been based on structures that map closely to theoretical tight-binding models, our TPCF, like other photonic crystals, has no direct tight-binding analog. Nonetheless, its photonic band structure can be shown to have nontrivial topology giving rise to disclination states. The Wannier centers are located on the sides of the periodic structure’s unit cell, as shown in the top plot of Fig. 1B, implying that adding a disclination binds fractional charge (*18*, *20*, *21*). Consistent with this prediction, numerical simulations of Maxwell’s equations on the structure of Fig. 1A (see text S2) reveal the existence of guided modes that are strongly localized to the center of the sample (Fig. 1E).

When light is coupled into the fabricated TPCF, we observe a spatially localized output profile at the end face (Fig. 1F). These optical microscope and infrared camera images are taken using a 100-m-long TPCF with a supercontinuum laser source coupled to the opposite end. Further details about the experimental setup are given in Materials and Methods. The light is concentrated at five high-index (glass) regions placed symmetrically around the central air hole, closely matching the prediction of Fig. 1E.

We will further investigate the disclination states, and their relationship to TCI topology, in the next section. It can be noted that reversing the large and small air holes yields a structure with trivial TCI topology, for which the Wannier centers lie at the centers of the unit cells (bottom plot of Fig. 1B). In that case, introducing a disclination will induce neither charge fractionalization nor defect states (see text S3).

The measured transmittance spectrum of the TPCF (Fig. 1G, top plot) shows that it can operate across a substantial wavelength band of around 700 to 1200 and 1500 to 1650 nm. We also determine the loss spectrum (Fig. 1G, bottom plot) by comparing the outputs for TPCFs with a length of 67 and 15 m, thus normalizing away the source spectrum (see Materials and Methods). We find an average loss of around 10 dB/km over 1000 to 1200 nm and around 20 dB/km over 1530 to 1625 nm. Although we have not yet performed rigorous optimization of the TPCF to minimize transmission losses, the current performance is already close to the level in commercial solid-core PCFs of similar core size (e.g., around 8 dB/km at 1064 nm for Thorlabs LMA-25, which has a core size of 25 μm).

### Guided topological defect modes

**Fig. 2.**
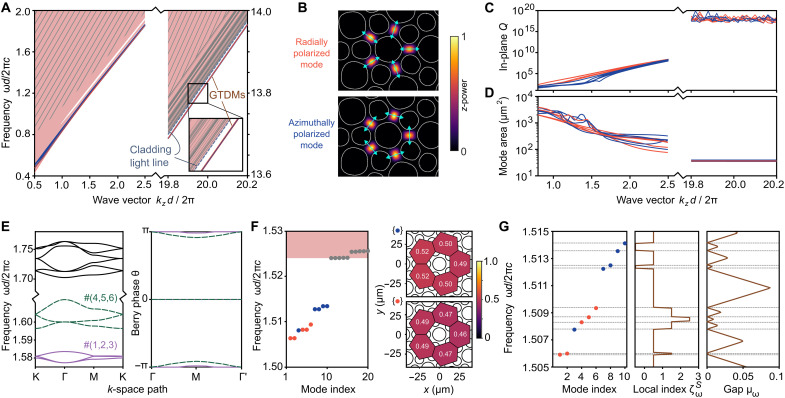
Analysis of GTDMs. (**A**) Band diagram for the measured TPCF structure (Fig. 1D). The GTDMs are plotted in red (radially polarized) and blue (azimuthally polarized), and bulk band frequencies are drawn as pink areas. In the large-$k_{z}$ regime on the right, dispersion curves for individual bulk states are plotted in gray. The gray-stroked region contains numerous modes that cannot be resolved numerically. (**B**) Calculated intensity profiles (normalized power flow in *z*) for exemplary radially and azimuthally polarized GTDMs at $k_{z}d/2\pi =20$ . Polarization directions are indicated by cyan arrows. (**C**) In-plane quality (*Q*) factors of the GTDMs versus $k_{z}$ . (**D**) Mode areas of the GTDMs versus $k_{z}$ . (**E**) Characterization of bulk TCI bands at fixed $k_{z}$ . Left: Band spectrum for the periodic TCI structure (corresponding to Fig. 1B, top plot). Right: Berry phases of the Wilson loop operator for different base points, using bulk bands #(1, 2, 3) and #(4, 5, 6). (**F**) Calculated eigenfrequencies for the preform hole profile (left; pink areas denote bulk bands), and the corresponding spectral charges in the five unit cells around the center (right). Blue/red dots respectively indicate azimuthally/radially polarized GTDMs. For details, see text S3. (**G**) Topological characterization via the spectral localizer (see text S4). Left: Eigenfrequencies of a symmetrized structure based on the preform profile. Middle: Local index $\zeta_{\omega }^{S}$ , where *S* is mirror symmetry around the *x* axis. Right: Local gap measure $\mu_{\omega }$ . The results for (E) to (G) are calculated at $k_{z}d/2\pi =2$.

Like most other PCFs, the TPCF, considered as a 3D structure, lacks a complete photonic bandgap. Using its measured cross-sectional structure (Fig. 1D), we calculate the transverse eigenmodes at each axial wave number $k_{z}$ , obtaining the band diagram shown in Fig. 2A (for details about the eigenmode calculations, see text S2). There are numerous bulk modes (pink-shaded regions) and a small number of disclination states that are strongly localized in the cross-sectional plane (red-and-blue lines), which we call GTDMs. Over some ranges of $k_{z}$ on the order of 2π/d , there are gaps in the transverse spectrum, but the GTDMs do not lie within them. Although the bulk-defect correspondence predicts the disclination states, it does not require them to lie within a gap and there is no chiral-like symmetry pinning their frequencies (*17*, *48*). Absent fine-tuning, disclination states in previous models have shown a similar tendency to migrate into the bulk state continuum (*20*, *21*).

The eigenmode calculations reveal a total of 10 GTDMs. With increasing $k_{z}$ , they dive below the “cladding light line” defined by the lowest-frequency bulk fiber modes. This feature is reminiscent of the “scalar limit” of guided modes in solid-core PCFs (*6*) and protects the GTDMs from coupling to bulk modes, similar to being in a bandgap. In the large-$k_{z}$ regime, the GTDMs become effectively degenerate in frequency, as shown on the right side of Fig. 2A; for reference, $20≲k_{z}d/2\pi ≲30$ corresponds to the 730- to 1100-nm operating regime of our experiments. The GTDMs form two groups of five modes each: one group has in-plane electric fields polarized in the radial direction, whereas the other is azimuthally polarized (Fig. 2B). Their intensities are strongly localized to five high-index regions surrounding the central air hole, similar to the previous ideal case (Fig. 1E). Moreover, with increasing $k_{z}$ , they exhibit rising in-plane quality (*Q*) factors and decreasing mode area (Fig. 2, C and D).

The location of the GTDMs below the cladding light also ensures that they have the lowest losses among all the guided modes of the TPCF. As a consequence, when modes are launched in the fiber through standard butt-in coupling (as discussed in the next section), the other modes will be rapidly damped with propagation distance, so that only the GTDMs remain nonnegligible. This desirable feature removes the need to couple to the GTDMs via precise focusing of the input light.

To understand the topological origins of the GTDMs, we return to the underlying 2D TCI (Fig. 1B, top plot), which has undeformed circular air holes and is spatially periodic (disclination-free). Its bands have zero Chern numbers, even for $k_{z}\neq 0$ , and are thus Wannier representable (*19*). We plot the bulk spectrum of this periodic structure for $k_{z}d/2\pi =2$ (Fig. 2E, left); a low $k_{z}$ is chosen so that the bands are more easily distinguishable. From this, we observe that the two lowest sets of bands, denoted by #(1,2,3) and #(4,5,6) , are separated from the others by gaps. We then analyze these bands using established methods for characterizing 2D TCIs (*18*, *19*, *21*). By examining the phase profiles of their Bloch wave functions at high-symmetry momentum points, we are able to determine that both sets of bands have symmetry indicators $\left(\chi_{M},\chi_{K}\right)=\left(2,0\right)$ (*18*, *19*, *21*). Then, by varying the base point of a Wilson loop, we observe nontrivial Berry phases around ±π (Fig. 2E, right), which corresponds to the Wannier centers being localized to the sides of the unit cell (Fig. 1B, top plot) (*21*). For more details about the characterization procedure, see text S3 and fig. S2. The results of the analysis imply that each set of bands binds fractional spectral charge, which is a necessary condition for localized topological states to emerge (*20*). To verify this, we turn to the preform hole profile (i.e., a finite structure containing a disclination), identify the GTDMs (Fig. 2F, left), and calculate their spectral charges. For each polarization, we find a charge of ≈0.5 in each of these five areas (Fig. 2F, right), in agreement with the TCI symmetry indicators. Note that an analogous tight-binding model has five disclination states arising from three bands (*21*), so the existence of 10 GTDMs can be interpreted as a doubling due to the polarization degree of freedom. The above properties also hold at the larger $k_{z}$ values where the TPCF operates.

To further confirm that the GTDMs are topological modes, we use an independent characterization framework called the spectral localizer (*41*). By combining information about a system’s position operators and Hamiltonian, the spectral localizer characterizes the system’s topology directly in real space. This approach complements the spectral charge analysis because it does not refer to a periodic precursor lattice and can accommodate the breaking of the TCI’s protecting symmetries by the disclination and fabrication-induced deformations. In the same spirit as topological band theory, the spectral localizer uses homotopy arguments to characterize a structure, e.g., by assessing through a topological invariant whether its Hamiltonian can be continued to a trivial insulator. In this case, the characterization is performed via (i) an index $\zeta_{\omega }^{S}$ and (ii) a local gap measure $\mu_{\omega }$ . At each frequency ω , $\zeta_{\omega }^{S}$ classifies what kind of atomic limit the system is continuable to while preserving a local spectral gap and a stated symmetry S ; we choose S to be a global mirror symmetry y→−y (the $C_{6v}$ symmetry of the precursor lattice is not usable as it is broken by the disclination). The values of $\zeta_{\omega }^{S}$ can be integers or half-integers, which correspond to distinct topological crystalline phases that cannot be continuously deformed into each other without breaking S and/or closing the local gap; changes in $\zeta_{\omega }^{S}$ are quantized to integers and correspond to the number of topological states at frequency ω (*44*). Meanwhile, $\mu_{\omega }$ quantifies the degree of topological protection, in the sense that a topological state at ω is robust against perturbations δH for which $\left\|\mathrm{\delta H}\right\|<\mu_{\omega }$ , where ∥⋅∥ denotes the largest singular value (*45*). For details, see text S4.

The spectral localizer analysis reveals that the 10 GTDMs are all associated with jumps in $\zeta_{\omega }^{S}$ (Fig. 2G, left and middle), alongside nonzero $\mu_{\omega }$ at intermediate frequencies (Fig. 2G, right). These results confirm that the GTDMs are robust topological modes. Furthermore, by extending the spectral localizer analysis using a generalized local gap measure (*47*), we are able to show that the results remain valid even if the TPCF is bent along an arbitrary direction (which deform the structure in a manner that need not preserve the mirror symmetry S , as explained in the next section); for details, see text S5.

### Characterization of fiber properties

Having established the existence of GTDMs in TPCFs and their topological origins, we show that their properties are well suited for waveguiding applications.

**Fig. 3.**
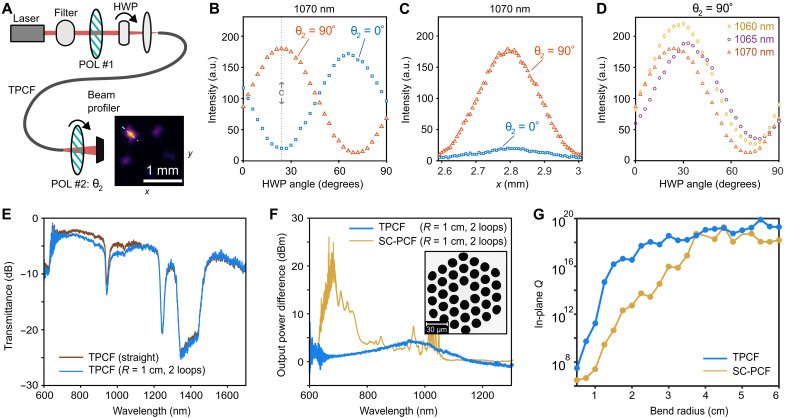
Polarization dependence and bending resistance. (**A**) Experimental setup with a tunable filter, fixed linear polarizer, and rotatable HWP between the source and the input face of a 0.5-m TPCF. A second linear polarizer, with variable angle $\theta_{2}$ , is placed between the end face and a beam profiler. Inset: Measured output intensity profile concentrated at one of the high-index regions around the central air hole. (**B**) Measured intensity at the center of the selected spot versus the HWP angle. Results are shown for two values of $\theta_{2}$ differing by 90°, with a fixed wavelength of 1070 nm. a.u., arbitrary units. (**C**) Variation of intensity with position, measured along a radial line passing through the selected spot [cyan dashes in the inset of (A)], with the HWP fixed at 24° [vertical dashes in (B)]. (**D**) Intensity at the center of the selected spot versus the HWP angle, for three different input wavelengths and fixed $\theta_{2}$ = 90°. (**E**) Measured transmittance for a straight TPCF (brown) and a TPCF with a two-loop bend with a radius of 1 cm (blue). (**F**) Output power difference between a straight fiber and one with a two-loop bend of radius 1 cm, for the TPCF (blue) and a comparable solid-core (SC) PCF (yellow). Inset: Scanning electron microscope image of the SC-PCF. (**G**) Calculated mean in-plane *Q* factors for the GTDMs in the TPCF (blue) and the fundamental core modes in the SC-PCF (yellow), for different bending radii at $k_{z}d/2\pi =30$ (corresponding to ~740 nm). For the SC-PCF in (F) and (G), the ratio between the air hole radii and the pitch is 0.38.

First, we verify that the theoretically predicted features of the GTDMs, including their aforementioned degeneracy, polarization, and spatial structure, are preserved during actual light transmission through the TPCF. Using the setup shown in Fig. 3A, we couple linearly polarized light at a single wavelength (1070 nm) into a 0.5-m-long TPCF. By adjusting the position of the beam spot on the input face of the TPCF, we find that the intensity profile at the end face can be strongly concentrated onto each of the five high-index regions around the central air hole (an example is shown in the inset of Fig. 3A). This is consistent with the degeneracy structure of the GTDMs, which allows for a choice of basis functions that break the structure’s fivefold rotational symmetry and localize on each of the five symmetry axes. Next, we use a half-wave plate (HWP) to rotate the input polarization angle, with another linear polarizer at relative angle $\theta_{2}$ placed at the end face (see Materials and Methods). As the HWP is rotated by 90° (which causes the input polarization to rotate by 180°), the output intensity varies sinusoidally over one cycle, and this curve shifts by a half-cycle if $\theta_{2}$ increases by 90° (Fig. 3B). We then fix the HWP at an extremal intensity setting (vertical dashes in Fig. 3B) and measure the intensity along a radial line passing through the output spot (cyan dashes in the inset of Fig. 3A), obtaining the profile shown in Fig. 3C. We hence conclude that the polarization of the GTDM is preserved as it passes through the TPCF. Moreover, upon tuning the wavelength filter, we find that this property is relatively insensitive to the operating wavelength (Fig. 3D).

A guided mode in a PCF should also be robust against coupling to cladding modes (because those can, in turn, couple to free space). The GTDMs are advantageous in this respect as they are tied to spectral charges originating from topological band invariants that do not change continuously when the system is weakly perturbed (*15*, *16*). The bulk-defect correspondence demands that this charge be localized to the disclination center, thereby obstructing mode delocalization via the hybridization of GTDMs with bulk states. This reasoning is also consistent with the nonzero local gap and index jump revealed by the spectral localizer (see figs. S3 and S4). Although such “topological protection” is never absolute (e.g., it can be spoiled by finite-size effects), the TPCF evidently operates in a regime where the GTDMs couple very weakly to other modes, as seen in the sizable gap between their dispersion relation and the cladding light line (Fig. 2A) and their strong spatial localization (Fig. 1E).

Accordingly, we expect the TPCF to perform well even when physically deformed. To test this, we subject a 90-m-long TPCF to two-loop bend, with 1-cm bending radius. Over much of the operating wavelength range, we find that the transmittance is only slightly reduced relative to the straight TPCF (Fig. 3E).

For comparison, we fabricate a solid-core PCF with similar core size and an air hole radii/pitch ratio of 0.38 (Fig. 3F, inset). For each fiber type, we measure the difference in output power (in dBm) between the straight and bent fiber (again using a two-loop bend with a radius of 1 cm). The solid-core PCF is found to have much stronger bending losses, by up to 25 dBm, particularly in the 600- to 800-nm range (Fig. 3F). To help understand these results, we perform numerical simulations in which the bend is modeled with a conformal transformation of the refractive index profile (see text S2). For the TPCF, the calculated *Q* factors for the GTDMs remain almost the same with decreasing bending radius, down to a radius of around 2 cm; by contrast, the solid-core PCF mode’s *Q* declines quickly as the bending radius goes below 4 cm (Fig. 3G).

## DISCUSSION

We have realized a TPCF supporting efficient broadband transmission that remains robust under strong bending. The design is based on a TCI hosting localized disclination states due to a bulk-defect correspondence. Such topological states have never previously been implemented in optical fibers, despite having been studied in the context of photonic crystal slabs (*20*, *21*, *34*). They turn out to have specific features that are advantageous for fiber waveguiding. In particular, the fact that they can exist outside bandgaps, previously regarded as a relatively obscure quirk (*20*, *21*), now enables the guided modes to reside below the cladding light line and thus decouple from the bulk modes. We have presented experimental results indicating that the TPCF is more robust to bending losses than a comparable solid-core PCF based on a conventional design. This calls for further quantitative studies, including comparisons to other fiber types as well as optimizations to our TPCF design, to determine whether topological modes are advantageous for optical fibers. It would also be interesting to explore alternative TPCF structures, including those using different kinds of band topology; many of the frameworks we have used to analyze our TPCF, especially the spectral localizer, may be useful for guiding such work.

The built-in degeneracy of the topological modes in our TPCF offers particularly intriguing possibilities for further research. In the future, it should be possible to develop more optimized procedures for selectively addressing the ten degenerate topological modes, for the purposes of spatial division multiplexing (*52*), or studying their interaction with nonlinear effects in fibers (*13*). It may also be possible to perform braiding on the degenerate topological modes, which would allow information to be encoded in their associated holonomy (*53*).

While this manuscript was in submission, we noticed a work demonstrating topological states in a helically twisted fiber (*9*). Unlike the disclination states used in this work, those topological states are edge states circulating around the boundary of the fiber core (*54*).

## MATERIALS AND METHODS

### Experimental setup

The optical fibers are illuminated using a supercontinuum laser (YSL Photonics SC-PRO 7 Supercontinuum Source; wavelength range of 430 to 2400 nm; peak bandwidth of 1050 to 1080 nm). The laser light passes through a lens and couples into the fiber head, which is fixed on a 3D stage. To optimize the input coupling to the fiber core, the fiber tail is connected to a power meter and the 3D stage is moved along three dimensions until the measured signal reaches a maximum. The power meter is then replaced by an optical signal analyzer (Yokogawa AQ6370C; wavelength range of 600 to 1700 nm) to record the output spectrum or a camera beam profiler (Thorlabs BC106N-VIS/M; wavelength range of 350 to 1100 nm) to record the mode profile.

To obtain the fiber loss spectrum (Fig. 1G, bottom plot), we follow a standard cut-and-measure procedure. The output spectrum is first measured for a TPCF with a length of 67 m. The fiber is then cut to a length of 15 m, and the output spectrum is measured again. The two spectra, expressed in logarithmic units, are subtracted from each other and the result divided by the truncated length to give the loss spectrum. The fiber transmittance (Fig. 1G, top plot) is obtained by taking the ratio of the TPCF output power to the output power from a multimode fiber (MMF) launched by the same source. The two output powers are expressed in linear scale, and the MMF output is self-normalized. This transmittance is then converted to a logarithmic scale and plotted in units of dBm.

When characterizing the polarization of the transmitted light (Fig. 3, A to D), a tunable filter (Fianium LLTF Contrast SWIR; wavelength range of 1000 to 2300 nm) is placed right after the source. By adjusting the 3D stage on which the fiber head is mounted, we locate a setting in which the output intensity is concentrated on one of the five high-index regions (Fig. 3A, inset), and we then use this to obtain the results in Fig. 3 (B to D). The reference angle $\theta_{2}$ is arbitrarily chosen but is fixed during all subsequent measurements. The intensities are directly extracted from the beam profiler.

In the bending loss experiment (Fig. 3, E and F), we place a 90-m-long fiber on a bending base with a preset bending radius. The other experimental procedures are as previously stated.

## Supplementary Materials

### This PDF file includes:

Supplementary Texts S1 to S5 Figs. S1 to S4 References
