# Supplementary material for: Topological photonic crystal fiber
Source: Sci Adv. 2025 Nov 12;11(46):eady1476. doi: 10.1126/sciadv.ady1476 (PMC12609044; doi:10.1126/sciadv.ady1476)
Supplement: Supplementary file 1 — Supplementary Texts S1 to S5 Figs. S1 to S4 References [file sciadv.ady1476_sm.pdf]

Science Advances
AAAS

Supplementary Materials for

Topological photonic crystal fiber

Bofeng Zhu *et al.*

Corresponding author: Qi Jie Wang, qjwang@ntu.edu.sg; Wonkeun Chang, wonkeun.chang@ntu.edu.sg; Yidong Chong, yidong@ntu.edu.sg

Sci. Adv. **11**, eady1476 (2025)
DOI: 10.1126/sciadv.ady1476

This PDF file includes:

Supplementary Texts S1 to S5
Figs. S1 to S4
References

Supplementary Text

S1. Design and fabrication details

To design the TPCF structure, we begin with a wedge of opening angle $\pi/3$ extracted from the periodic TCI structure (Fig. S1A). This has circular air holes of alternating radii $0.57d$ and $0.35d$, where d is the center-to-center distance between neighbouring holes. We deform this into a $2\pi/5$ wedge by scaling each site's azimuthal coordinate by $6/5$ (Fig. S1B). Copying this wedge to the remaining lattice sectors yields the structure of Fig. 1A, featuring a disclination of Frank angle $-\pi/3$.

Next we make small adjustments to the site positions, with the aim of improving the overall spatial uniformity of the PCF structure. This is achieved with the aid of a molecular dynamics (LAMMPS (*55*)) simulation, which moves a set of "atoms" centered at the site positions while also gradually enlarging their radii, subject to the fixed boundaries of the wedge (*56*). This yields a jam-packed configuration (Fig. S1C), in which the atomic radii ($R''_1 = 0.65d$ and $R''_2 = 0.43d$) correspond to the outer surfaces of the glass capillaries we aim to use in the fiber preform (see below). Finally, we define the air holes by downscaling the radii to $R'_1 = 0.49d$ and $R'_2 = 0.33d$, corresponding to the inner surfaces of the capillaries. The resulting structure (Fig. S1D) corresponds to the arrangement shown in the inset of Fig. 1C.

To fabricate the fiber, we draw glass tubes of outer (inner) diameter 25 mm (19 mm) into two sets of smaller tubes, or capillaries. After adjusting the drawing conditions (temperature, vacuum pressure, etc.), we obtain capillaries with outer diameters $R'_1 = 3.33$ mm (11 pieces) and $R'_2 = 2.18$ mm (20 pieces), close to the ideal ratio described above. Additional glass tubes of outer (inner) diameter 10 mm (3 mm), composed of the same silica material as the capillaries, are drawn into solid rods with collapsed inner air holes and outer diameters of 1.095 mm (5 pieces), 0.702 mm (20 pieces), 1.619 mm (15 pieces), and 1.905 mm (10 pieces). The capillaries and rods are stacked in a jacket of outer (inner) diameter 25 mm (19 mm) according to the arrangment in the inset of Fig. 1C, with the rods filling the major gaps between the capillaries. The preform is drawn into a preform cane of diameter 4.7 mm (Fig. 1D), which is in turn drawn into the TPCF in a fiber drawing tower.

The drawing process introduces deformations to the dielectric structure. From a scanning

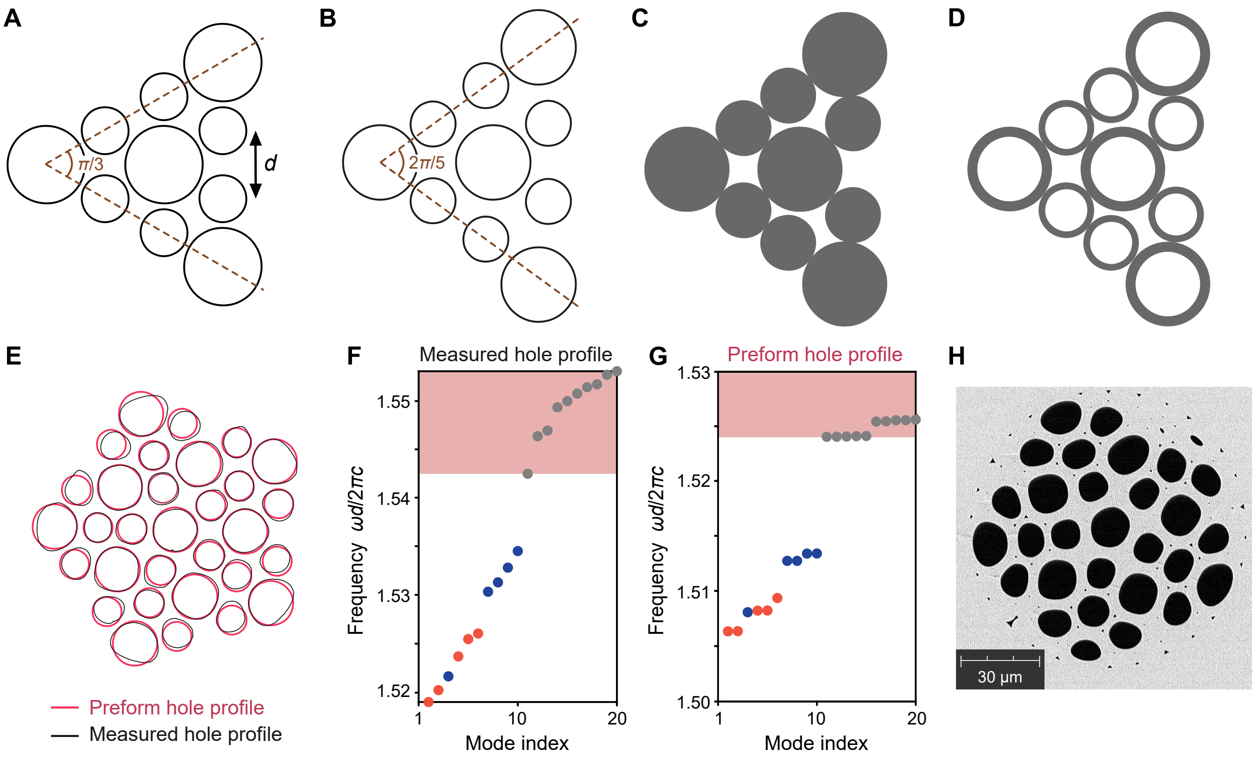

Figure S1: **Lattice design.** (**A**) Wedge with opening angle $\pi/3$, extracted from a triangular lattice with nearest neighbor center-to-center spacing d. The discs (air holes) have alternating radii of $0.57d$ and $0.35d$. (**B**) Wedge with opening angle $2\pi/5$, generated from (A) by uniformly scaling the azimuthal coordinates of the site centers. This corresponds to the structure shown in Fig. 1A. (**C**) Wedge generated from (B) by gradually enlarging the disc radii and adjusting their positions, with boundaries fixed, until they are jam-packed. (**D**) Structure generated from (C) by assigning inner air holes of radius $R_1' = 0.49d$ and $R_2' = 0.33d$ to the discs. This corresponds to the target stacking arrangement shown in Fig. 1C. (**E**) Comparison between the preform hole profile based on (D) (black lines), and the measured hole profile of the fabricated TPCF (pink lines). The air holes radii are slightly enlarged to $R_1 = 0.55d$ and $R_2 = 0.36d$. (**F** and **G**) Calculated eigenfrequencies at $k_z d/2\pi = 2$ for the measured hole profile (F) and the preform hole profile (G). Red (blue) data points correspond to GTDMs with radial (azimuthal) polarization, while gray data points are bulk states. The pink-shaded region indicates the bulk band. (**H**) Scanning electron microscope image of the fiber cross section. The small black specks correspond to non-filled interstitial air holes, which can be seen to mostly occur in the out-of-core region.

electron microscope image of the fiber end face (Fig. 1D), we find that the holes become non-circular, but their positions are relatively unaffected (Fig. S1E). The air hole radii are also slightly enlarged to $R_1 = 0.55d$ and $R_2 = 0.36d$, which are the values used in the preform hole profile for numerical simulations (see below). From numerical calculations of the eigenmodes (see below), we find that the deformations induce small shifts in the frequencies of the GTDMs and bulk states, but do not alter the qualitative features of the spectrum (Fig. S1F and G). We also observe that some of the interstitial air holes fail to be completely filled-in, resulting in some small holes that are mainly concentrated in the out-of-core areas (Fig. S1H); we expect that these imperfections can be eliminated in the future by optimizing the drawing conditions, but they are in any case unlikely to influence the GTDMs, which are localized near the core.

We use the TPCF's measured hole profile (i.e., the black lines in Fig. S1E) for most of our numerical calculations, including the band diagram (Fig. 2A), various GTDM properties (Fig. 2B–D), bending performance calculation (Fig. 3G) and 2D spectral localizer calculations (Fig. S4). The exception is the spectral charge calculation (Fig. 2F), which uses the preform hole profile, and the 1D spectral localizer calculations (Fig. 2G and Fig. S3B), which use the preform hole profile with a mirror symmetry-breaking perturbation (see below).

The conventional solid-core PCF is fabricated via the same stack-and-draw process, using a single set of glass tubes with outer (inner) radius 20 mm (16 mm) drawn into capillaries of outer diameter 2.28 mm (36 pieces). A single solid rod of 2.28 mm is used for the core. The hexagonal preform is supported in the cylindrical jacket by solid rods of outer diameter 0.61 mm (6 pieces), 0.75 mm (30 pieces) and 0.97 mm (12 pieces).

S2. Calculating photonic eigenmodes and band diagrams

An optical fiber is translationally symmetric in the axial (z) direction. For a given axial wavenumber (k_z), the electromagnetic eigenmodes can be determined by solving Maxwell's equations in the 2D (x-y) plane. In this work, we perform the calculations numerically via the finite-element method (FEM) simulation software COMSOL Multiphysics, solving the full vectorial form of Maxwell's equations with no additional approximations apart from the discretization of space. The principal input to this calculation is the real-valued dielectric profile $\epsilon(\mathbf{r})$, where $\mathbf{r} = (x, y)$ is the position in the 2D plane. We model the structure's high-index (silica) and low-index (air) regions with $\epsilon = 2.1$

and $\epsilon = 1$ respectively.

Each calculated eigenmode μ has some angular frequency ω_μ and electric field profile

$$\mathbf{E}_\mu(\mathbf{r}) \equiv \langle \mathbf{r} | \mu \rangle, \tag{S1}$$

which is a complex 3-vector-valued field defined in the 2D space; from this, the full physical electric field is given by $\mathrm{Re}\left[\mathbf{E}_\mu(\mathbf{r}) \exp(ik_z z)\right]$. Both ω_μ and $\mathbf{E}_\mu(\mathbf{r})$ depend implicitly on k_z. The inner product between two normalized eigenmodes is defined as (*10*)

$$\langle \mu | \nu \rangle = \int d^2r \; \epsilon(\mathrm{r}) \; \mathbf{E}_\mu^*(\mathbf{r}) \cdot \mathbf{E}_\nu(\mathbf{r}). \tag{S2}$$

If the boundary conditions are Hermitian (e.g., Dirichlet or periodic boundary conditions), Maxwell's equations consitute a Hermitian generalized eigenproblem, so the orthogonality relation $\langle \mu | \nu \rangle = \delta_{\mu\nu}$ holds (*10*).

When simulating the spatially finite TPCF structure, our choice of boundary conditions depends on the circumstances. For the spectral localizer (Fig. 2G and Fig. S3B) and finding the spectral charges (Fig. 2F), it is important that the eigenproblem be Hermitian, so we apply perfect electric conductor (PEC) boundary counditions to the system's exterior boundary. In all other cases, including calculations of the mode profiles (Fig. 1E and Fig. 2B), dispersion relations (Fig. 2A) and the 2D localizer calculations (Fig. S4), we apply impedance boundary conditions, which are equivalent to an infinite external medium of $\epsilon = 2.1$. Since light can leak out of the fiber and escape to infinity, the eigenfrequencies become complex; the real parts are used in the dispersion relations (Fig. 2A), and the imaginary parts are used to determine Q factors (Fig. 2C and Fig. 3G). From the field distributions, we can also extract the effective mode areas (Fig. 2D), which are defined as (*3*)

$$A = \frac{\left(\int d^2r |E_\mu(\mathbf{r})|^2 \right)^2}{\int d^2r |E_\mu(\mathbf{r})|^4}, \tag{S3}$$

where the eigenmode $E_\mu(\mathbf{r})$ has been normalized using the inner product Eq. (S2).

When modeling the effects of bending the fiber (Fig. 3G and Fig. S4), we perform a conformal transformation on the refractive index profile (*62*). The modified refractive index (the square root of the dielectric constant) is

$$n'(x, y) = n(x, y) \left(1 - \frac{x}{R}\right), \tag{S4}$$

where $n(x, y)$ is the refractive index distribution for the straight fiber with R as the bending radius. The ideal preform hole profile (Fig. S1E) with such index modulation is used for the spectral localizer calculations presented in Fig. 2G and Fig. S3B. We use the measured hole profile, which lacks a mirror symmetry, for the additional spectral localizer analysis based on local gap profiles in Fig. S4.

When analysing the underlying photonic crystal structure (Fig. 1B), which is spatially infinite and periodic in the x-y plane, we impose Bloch's theorem by writing

$$\mathbf{E}_{n,\mathbf{k}}(\mathbf{r}) = \mathbf{u}_{n,\mathbf{k}}(\mathbf{r})\; e^{i\mathbf{k}\cdot\mathbf{r}}, \tag{S5}$$

where $\mathbf{k}$ is a 2D wave-vector (quasimomentum) and n is a band index. Such eigenfunctions are calculated numerically using a single unit cell with Floquet-Bloch boundary conditions. The eigenproblem is Hermitian, with inner products between Bloch states given by

$$\langle n, \mathbf{k} | n', \mathbf{k}' \rangle = \int_{\text{u.c.}} d^2 r\; \epsilon(\mathrm{r})\; \mathbf{u}^*_{n,\mathbf{k}}(\mathbf{r}) \cdot \mathbf{u}_{n',\mathbf{k}'}(\mathbf{r}), \tag{S6}$$

where the integral is taken over a single unit cell.

S3. Wannier centers and spectral charge

Like other TCIs, the C_6-symmetric precursor structure for our TPCF can be topologically characterized using symmetry indicators. In this section, we will explain the procedure, which largely follows earlier studies (*18, 19, 21*). It is worth noting that these methods are applicable even though the photonic structure we are considering is not based on a tight-binding model.

Given a set of bands that are well-defined (i.e., not overlapping with any other bands), one defines $\#\Pi_p^{(P)}$ as the number of bands at a high-symmetry momentum point $\Pi \in \{\Gamma, \mathrm{M}, \mathrm{K}\}$ that has the C_n rotation eigenvalue $e^{\mathrm{i}2\pi(p-1)/P}$ $(p = 1, \ldots, P)$. We can then calculate the topological index $(\chi_\mathrm{M}, \chi_\mathrm{K})$, where $\chi_\mathrm{M} = \#\mathrm{M}_1^{(2)} - \#\Gamma_1^{(2)}$ and $\chi_\mathrm{K} = \#\mathrm{K}_1^{(3)} - \#\Gamma_1^{(3)}$ (*18*).

From their Bloch function phase profiles, the topological index of bands $\#(1, 2, 3)$ is calculated by first calculating the $\Pi_p^{(n)}$ index for each individual band and then summing these indices across all three bands (see Fig. S2A),

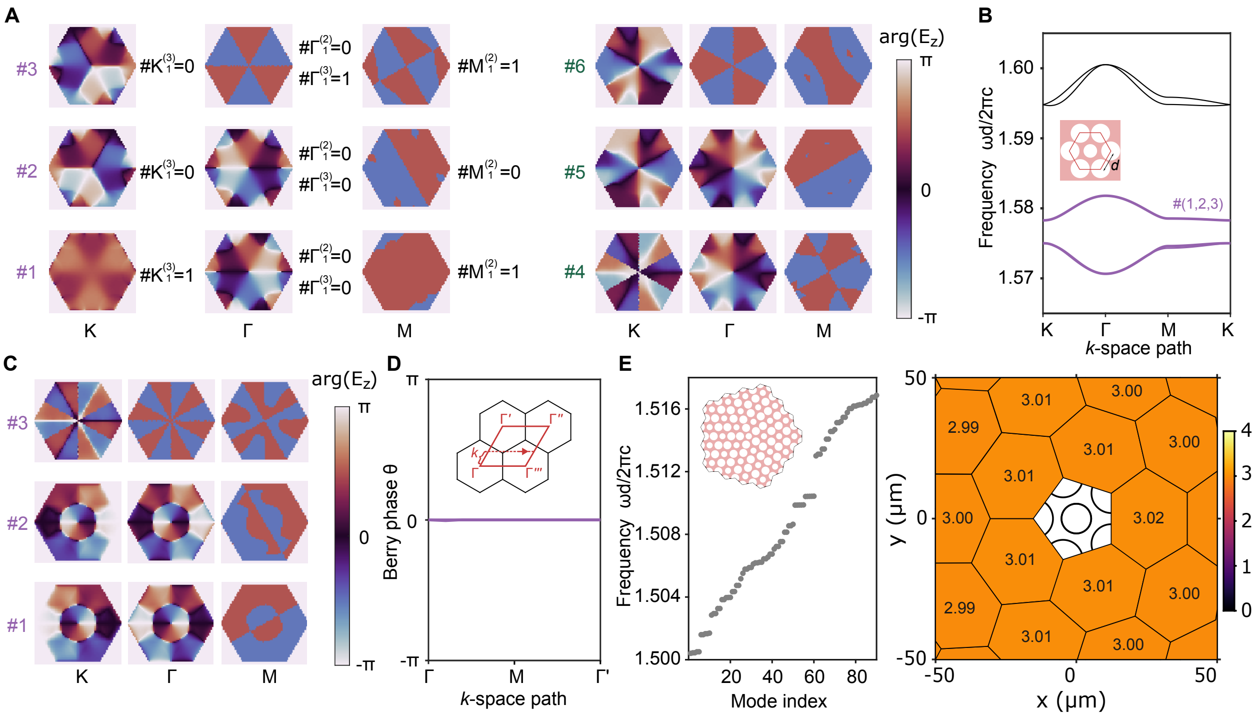

Figure S2: **Wilson loop and spectral charge calculations.** (**A**) Phase profiles of the out-of-plane electric field (E_z) for the six Bloch states marked in Fig. 2E at high-symmetry momentum points. The symmetry indicators of bulk bands #(1, 2, 3) are labeled on the right. (**B**) Lowest five bands for the periodic photonic structure with trivial crystalline topology, calculated from the unit cell in Fig. 1B (lower plot). (**C**) Phase profiles of the out-of-plane electric field (E_z) for the Bloch states #(1, 2, 3) in the trivial structure of (**B**) at high-symmetry momentum points. (**D**) Berry phases of the Wilson loop operator at different initial k for the trivial bulk bands #(1, 2, 3). Inset: the schematic of the Wilson loop. The base point lies along the path $\Gamma \rightarrow \Gamma'$, and the Wilson loop follows the dashed line running parallel to Γ–Γ'''. (**E**) Left panel: calculated eigenfrequencies for the trivial PCF structure, based on Fig. 2F but with large and small air holes switched. Inset: schematic of the lattice, composed of 30 intact unit cells and a central defect. Right panel: calculated spectral charges (black numbers) for the schematic structure in the left panel. In (**A–E**), the eigenmodes are calculated at $k_z d/2\pi = 2$. The spectral charge calculations are performed using symmetric preform hole profiles.

$$\#\Gamma_1^{(2)} = 0\,(0), \quad \#\Gamma_1^{(3)} = 1\,(1)$$
$$\#\mathrm{M}_1^{(2)} = 2\,(2) \quad \#\mathrm{K}_1^{(3)} = 1\,(1). \tag{S7}$$

The index of bands #(4, 5, 6) can be calculated in a similar way, and both sets of bands have index $(\chi_M, \chi_K) = (2, 0)$. The indicators of topological trivial case can be calculated from Fig. S2B and C, with a trivial index of $(\chi_M, \chi_K) = (0, 0)$.

The topological bulk-defect correspondence in such TCIs states that the symmetry indicators are related to defect charges of (*19, 21*)

$$\mathrm{Q_{dis}} = \frac{\Omega}{2\pi}\left(\frac{3}{2}\chi_{\mathrm{M}} - \chi_{\mathrm{K}}\right) \bmod 1, \tag{S8}$$

where $\Omega = -\pi/3$ is the Frank angle of the disclination. Applying this to our TCI, the two sets of bands, #(1, 2, 3) and #(4, 5, 6), are each predicted to produce fractional charge $\mathrm{Q_{dis}} = 1/2 \pmod 1$.

To verify the correspondence, we explicitly calculate the spectral charges. Even though there are two half-charges, we can distinguish them by exploiting the polarization structure of the GTDMs. For each polarization (radial or azimuthal), we pick out the five GTDMs and calculate

$$\sum_{u\,\in\,\mathrm{GTDMs}} \int_S d^2r\; \epsilon(\mathrm{r})\; \left|\mathbf{E}_\mu^*(\mathbf{r})\right|^2, \tag{S9}$$

where S is one of the five unit cells surrounding the core unit cell.

To determine the Wannier centers of the photonic TCI (Fig. 1B), we use the Wilson loop approach (*57, 58*). Given a set of degenerate bands and a path $k + l \leftarrow k$ passing across the Brillouin zone, we discretise the path into steps separated by $\Delta\mathbf{k}$ and calculate

$$W_{k+l\leftarrow k} = G^{k+l-\Delta k}\, G^{k+l-2\Delta k} \cdots G^{k+\Delta k}\, G^{k} \tag{S10}$$
$$G^k_{mn} = \langle\, m, \mathbf{k} \,|\, n, (\mathbf{k} + \Delta\mathbf{k})\,\rangle, \tag{S11}$$

where m, n are band indices and Eq. (S11) uses the inner product defined in Eq. (S6). To construct the path of the Wilson loop, we first consider a rhombus with corners at four adjacent Γ points in the extended Brillouin zone (inset of Fig. S2D). The initial k-point (or "base point") is swept along the path $\Gamma \to \mathrm{M} \to \Gamma'$, with the Wilson loop path taken parallel to the line $\Gamma \to \Gamma'''$.

The eigenvalue spectrum of the W operator is adiabatically deformable to a set of centers of localized Wannier functions in real space. As established in previous works (*58–60*), topologically

nontrivial TCIs have nonzero values of the Berry phase $\mathrm{Im}\{\log\left[\det\left(W_{k+l\leftarrow k}\right)\right]\}$ (Fig. 2E), corresponding to the Wannier centers not being located at the center of the unit cell. In contrast, the Berry phase of trivial TCI remains close to zero, as shown in Fig. S2D.

The calculation of spectral charge for the topological trivial case (Fig. S2E) is similar but the summation is done for all the 90 bulk states below the bandgap, which are contributed from the lowest three bulk bands, i.e., band #(1,2,3) in Fig. S2B, of all the 30 intact unit cells.

S4. Symmetry-reduced spectral localizer

The calculations in the previous section strongly indicate that the TPCF should host topological disclination states, but there are some limitations in the argument. First, those characterization frameworks refer to an underlying periodic bulk TCI, yet the symmetries on which the TCI relies are broken by the disclination and the various aforementioned deformations in the TPCF. Second, in a lattice exhibiting charge fractionalization, it is sometimes possible for a strong lattice disturbance, like the core of a disclination, to remove the topological states expected to accompany the fractionalization (*20*). Finally, even if the lattice hosts topological disclination states, a given disclination state can still be accidental, i.e., non-topological. To resolve these doubts, we turn to the spectral localizer framework (*41–44*), which provides a way to directly establish that the disclination states in the TPCF are topological in origin as well as robust.

For a system with a single relevant position operator Y_c, the spectral localizer characterizes the local topology at frequency ω and position y by combining Y_c and the system's Hamiltonian $H_{\mathrm{eff},c}(\omega)$ into

$$L_{(y,\omega)}(Y_c, H_{\mathrm{eff,c}}) = \begin{pmatrix} 0 & H_{\mathrm{eff,c}}(\omega) - i\kappa\left(Y_c - y\mathbf{1}_c\right) \\ H_{\mathrm{eff,c}}(\omega) + i\kappa\left(Y_c - y\mathbf{1}_c\right) & 0 \end{pmatrix}, \quad \text{(S12)}$$

where κ is a scaling coefficient used to make the units consistent and to balance the spectral weight between Y_c and $H_{\mathrm{eff,c}}$. The notation here follows Ref. (*46*). Both Y_c and $H_{\mathrm{eff},c}$ are numerically extracted from the discretized master equations in the FEM eigenfrequency solver—the same solver used all in our other numerical calculations. In particular, $H_{\mathrm{eff},c}$ is a matrix-valued function of the frequency ω, meaning the photonic eigenmodes correspond to eigenvectors of $H_{\mathrm{eff,c}}(\omega)$ with zero eigenvalue (*46*).

The TPCF structure possesses a mirror symmetry $M_y : y \longrightarrow -y$, which satisfies $M_y^2 = \mathbf{1}$, $H_{\text{eff},c} M_y = M_y H_{\text{eff},c}$ and $Y_c M_y = -M_y Y_c$. Using this symmetry, the reduced spectral localizer can be constructed as

$$L_\omega^{M_y}(Y_c, H_{\text{eff,c}}) = \left[H_{\text{eff,c}}(\omega) + i\kappa Y_c\right] M_y. \tag{S13}$$

Topological defect modes can then be characterized by a local marker defined as

$$\zeta_\omega^{M_y}(Y_c, H_{\text{eff,c}}) = \frac{1}{2}\text{sig}\left[L_\omega^{M_y}(Y_c, H_{\text{eff,c}})\right], \tag{S14}$$

where $\text{sig}[\cdots]$ denotes the signature (i.e., the difference between the total number of positive and negative eigenvalues). Note that Eq. (S13) is Hermitian, so its signature is well-defined.

The key idea is to use the local index (S14) to classify the topology of the system, in terms of what kind of atomic limit it is continuable to, at each frequency ω and subject to the specified symmetry M_y. For example, in a periodic lattice, one atomic limit would correspond to Wannier centers located at the center of the unit cell, while an obstructed atomic limit would correspond to Wannier centers located at the edges or corners of the unit cell.

If the value of $\zeta_\omega^{M_y}$ changes at a frequency ω, that implies that the modes at that frequency have non-trivial topology (*43*). Such a change happens when an eigenvalue of the spectral localizer crosses zero. Therefore, the local marker is also associated with a local measure of topological robustness via a "local gap",

$$\mu_\omega(Y_c, H_{\text{eff,c}}) = \min\left|\text{spec}\left[L_\omega^{M_y}(Y_c, H_{\text{eff,c}})\right]\right|, \tag{S15}$$

where $\text{spec}[\cdots]$ denotes the spectrum of the matrix.

We apply the spectral localizer directly on the finite element represention of the TPCF's photonic structure (see Section S3). When doing this, there are two important subtleties to handle. The first involves the boundary conditions encoded into the FEM matrices. The subscript c in $H_{\text{eff},c}$, Y_c and $\mathbf{1}_c$ refers to "eliminated matrices", which have undergone a procedure whereby all degrees of freedom involved in the boundary conditions are removed (*46*). This projection is realized using matrices denoted by "Nullf" and "Null", composed of basis vectors spanning the null space of the constraint force Jacobian matrix and the constraint Jacobian matrix, respectively: specifically, $H_{\text{eff},c} = \text{Nullf}^T H_{\text{eff}}\text{Null}$, $Y_c = \text{Nullf}^T Y \text{Null}$ and $\mathbf{1}_c = \text{Nullf}^T\text{Null}$, where H_{eff} and Y are matrices retrieved directly from the FEM (including boundary degrees of freedom).

The next subtlety involves the mirror symmetry. Even if the structure and the mesh are both symmetric with respect to M_y, the discretized FEM master equations may not yield a (non-eliminated) effective Hamiltonian H_{eff} commuting with M_y. This can be problematic since, as explained above, the local marker assumes mirror symmetry, which is necessary to guarantee the Hermiticity of Eq. (S13). To bypass this difficulty, we decompose H_{eff} into even and odd subspaces with respect to M_y,

$$H_{\text{eff}} = \begin{pmatrix} H_{\text{eff},+} & 0 \\ 0 & H_{\text{eff},-} \end{pmatrix}. \tag{S16}$$

The sub-Hamiltonians $H_{\text{eff},+}$ and $H_{\text{eff},-}$ are obtained from the FEM solver by applying symmetric/antisymmetric boundary conditions to the mirror line. In a similar way, we decompose the Nullf and Null matrices. In this basis, the mirror symmetry matrix reads

$$M_y = \begin{pmatrix} \mathbf{1}_+ & 0 \\ 0 & -\mathbf{1}_- \end{pmatrix}, \tag{S17}$$

where $\mathbf{1}_\pm$ are identity matrices of the same size as $H_{\text{eff},\pm}$. Finally, as the position operator Y anticommutes with M_y, we can write it as

$$Y = \begin{pmatrix} 0 & Y_- \\ Y_+ & 0 \end{pmatrix}, \tag{S18}$$

where Y_+ and Y_- are diagonal matrices constructed from the y coordinates of the symmetric and antisymmetric reduced systems.

We calculate the spectral localizer for a structure based on the ideal TPCF preform hole profile (Fig. S1E), which is mirror symmetric with respect to the x axis. To aid the analysis, a mirror-symmetric refractive index perturbation is introduced to break the degeneracy between the doublet states among the GTDMs; we opt for a perturbation consistent with fiber bending (Eq. (S4)). In Fig. 2G and Fig. S3, we use a bending radius of $R = 1.5$ cm, along with $y = 0$ and $\kappa = \kappa_0 \left[10^{-4}\|H_{\text{eff},c}(\omega_0)\|/\|Y_c\|\right]$. The local gap is normalized by $10^{-4}\|H_{\text{eff},c}(\omega_0)\|$, where $\|\cdots\|$ is the spectral norm (i.e., largest singular value). Moreover, Fig. 2G uses $\kappa_0 = 0.01$ and $\omega_0 = 1.5(2\pi c/d)$, while Fig. S3 uses $\kappa_0 = 0.1$ and $\omega_0 = 20.73(2\pi c/d)$.

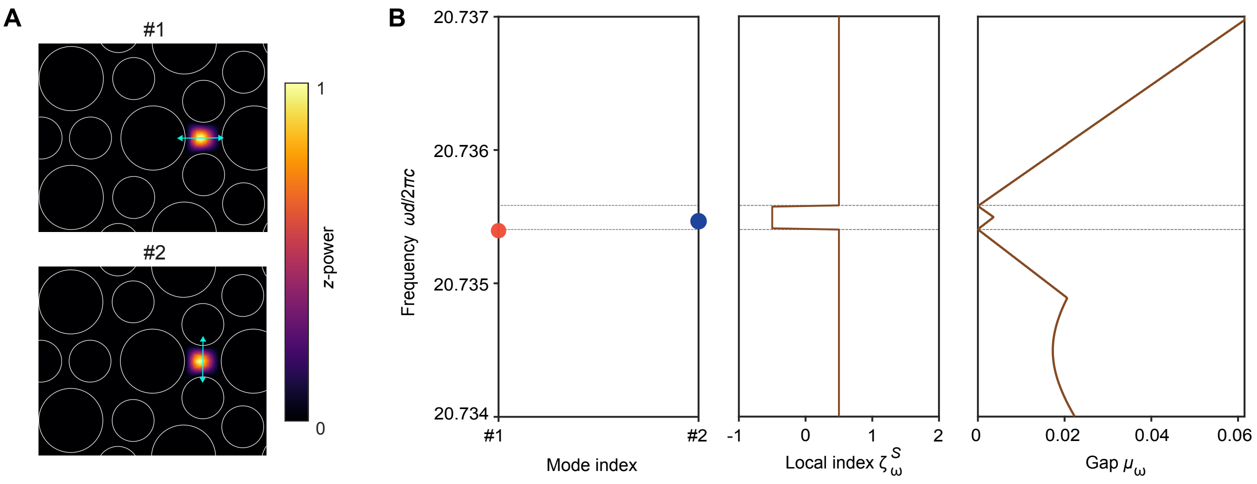

Figure S3: **Spectral localizer results for bent fiber.** (**A**) Calculated intensity profiles (normalized power flow in z) for a pair of radially and azimuthally polarized GTDMs. Polarization directions are indicated by cyan arrows. (**B**) Eigenmode spectrum (left panel), local index ζ^S_ω (center panel) and local gap μ_ω (right panel) from spectral localizer calculations. In these calculations, we use $k_z d/2\pi = 30$ and bending radius $R = 1.5$ cm.

S5. 2D localizer for bending analysis

The spectral localizer can also quantify how robust the GTDMs are to perturbations that do *not* obey the previously-assumed mirror symmetry. To accomplish this, we employ the 2D localizer (*47, 61*)

$$L_{x,y,\omega}(X_c, Y_c, H_{\text{eff,c}}) = \begin{pmatrix} H_{\text{eff,c}}(\omega) & \kappa\,(X_c - x\mathbf{1}_c) - i\kappa\,(Y_c - y\mathbf{1}_c) \\ \kappa\,(X_c - x\mathbf{1}_c) + i\kappa\,(Y_c - y\mathbf{1}_c) & -H^\dagger_{\text{eff,c}}(\omega) \end{pmatrix}. \tag{S19}$$

Unlike the 1D localizer in Eq. (S13), this can probe 2D positions (x, y) without assuming the structure to be mirror symmetric or the system's Hamiltonian to be Hermitian. We do not use this to formulate a local index, instead focusing on the local gap measure (also called a "linear local gap") (*47*),

$$\mu_{x,y,\omega}(X_c, Y_c, H_{\text{eff,c}}) = \min\left|\text{Re}\left(\text{spec}\left[L_{x,y,\omega}(X_c, Y_c, H_{\text{eff,c}})\right)\right]\right|, \tag{S20}$$

where spec$[\cdots]$ denotes the spectrum of the matrix. The condition $\mu_{x,y,\omega} \approx 0$ is associated with the existence of a localized state near (x, y) at frequency ω.

We can now determine whether these zeros remain at these positions, or move away, when the ideal fiber structure is perturbed (such as through bending).

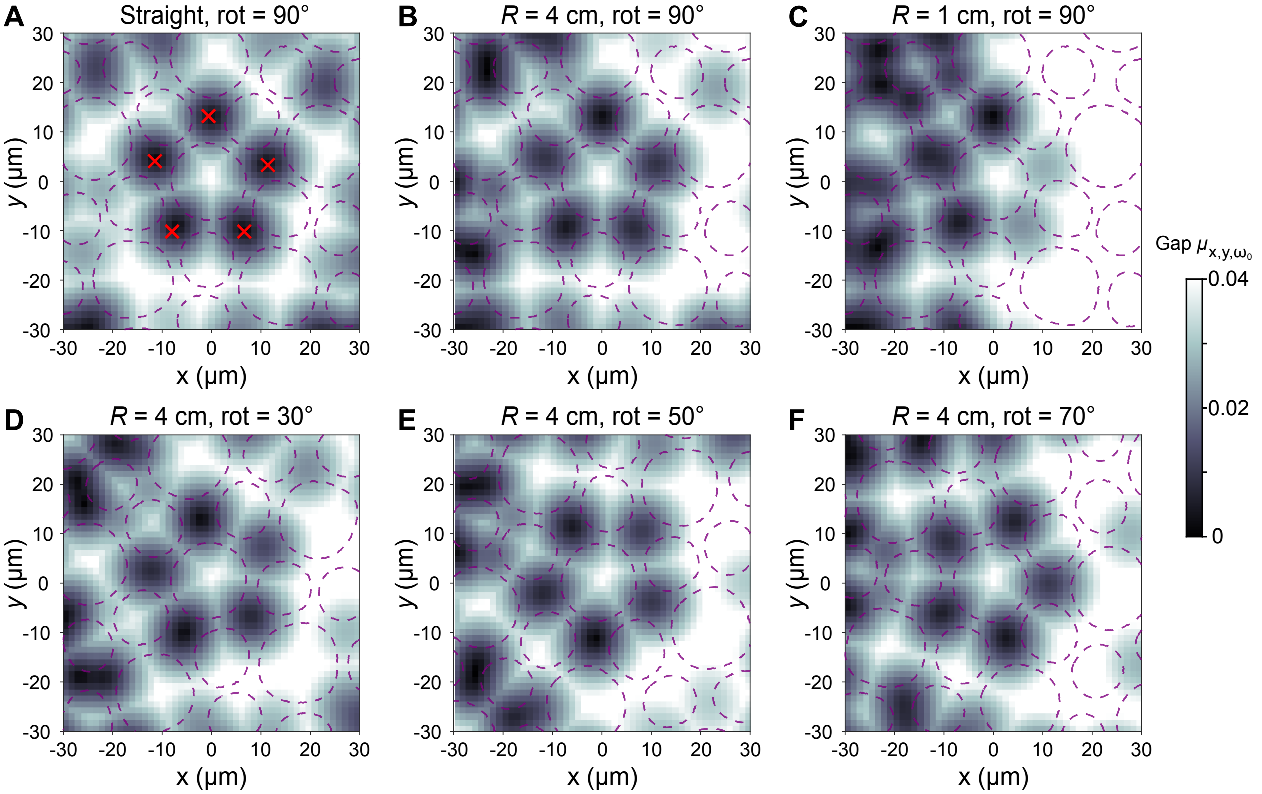

Figure S4: **Characterization of GTDM robustness by local gap.** (**A**) Local gap μ_{x,y,ω_0} calculated at $k_z d/2\pi = 30$ and $\omega_0 = 20.72(2\pi c/d)$ for the measured hole profile, consisting of the structure of Fig. S1E rotated counter-clockwise by 90°. The five zeros of μ_{x,y,ω_0} near the disclination core (red crosses) correspond to the GTDMs, previously identified as topological states via jumps in a local index (Fig. 2G). (**B** and **C**), Local gaps calculated at the same k_z and ω_0 for structures deformed by bending the fiber along x with bending radius $R = 4\,\text{cm}$ (B) and $R = 1\,\text{cm}$ (C). (**D—F**), Local gaps calculated at bending radius of $R = 4\,\text{cm}$, but from the measured hole profile rotated counter-clockwise by 30° (D), 50° (E) and 70° (F). This bending spoils the mirror symmetry previously assumed in the symmetry-reduced spectral localizer analysis.

We first consider the measured hole profile (see Fig. S1E) and show the corresponding $\mu_{x,y,\omega}$ in Fig. S4A. We indeed observe zeros of $\mu_{x,y,\omega}$ at the locations of the GTDMs (here we take $k_z d/2\pi = 30$, for which the GTDMs are near-degenerate), confirming the robustness of fiber design against fabrication distortions (*45*). In addition to taking into account the measured hole profile, we plot in Fig. S4B and C the local gap $\mu_{x,y,\omega}$ for the perturbed structures corresponding to bending the fiber in the x-axis, with bending radii of 4 cm and 1 cm respectively. The fiber structure is obtained by rotating the measured hole profile by 90° (counter-clockwise direction). The bending along the x-axis is simulated using a conformal transformation on the refractive index (in both dielectric and air) given Eq. (S4). When the bending becomes too large, e.g. for a bending radii of 1 cm in Fig. S4C, the zeros of $\mu_{x,y,\omega}$ associated with the GTDMs starts to merges with each others or with the bulk modes, indicating the break down of the GTDMs.

Bendings along arbitrarily axis are also considered by first rotating the measured hole profile with different angles and then applying a bending along the x-axis. Figures S4D and F show $\mu_{x,y,\omega}$ for the fiber rotated, respectively, by 30°, 50° and 70°, all for a given bending radius of 4 cm. Altogether, the zeros of $\mu_{x,y,\omega}$ associated with the GTDMs remain nearly unchanged around the fiber core, demonstrating the robustness of the GTDMs against fiber bending (*45*).

REFERENCES AND NOTES

1. J. D. Joannopoulos, P. R. Villeneuve, S. Fan, Photonic crystals: Putting a new twist on light. *Nature* **386**, 143–149 (1997).

2. J. C. Knight, Photonic crystal fibres. *Nature* **424**, 847–851 (2003).

3. C. Markos, J. C. Travers, A. Abdolvand, B. J. Eggleton, O. Bang, Hybrid photonic-crystal fiber. *Rev. Mod. Phys.* **89**, 045003 (2017).

4. E. N. Fokoua, S. A. Mousavi, G. T. Jasion, D. J. Richardson, F. Poletti, Loss in hollow-core optical fibers: Mechanisms, scaling rules, and limits. *Adv. Opt. Photonics* **15**, 1–85 (2023).

5. B. Temelkuran, S. D. Hart, G. Benoit, J. D. Joannopoulos, Y. Fink, Wavelength-scalable hollow optical fibres with large photonic bandgaps for CO_2 laser transmission. *Nature* **420**, 650–653 (2002).

6. T. A. Birks, J. C. Knight, P. St. J. Russell, Endlessly single-mode photonic crystal fiber. *Opt. Lett.* **22**, 961–963 (1997).

7. C. M. Smith, N. Venkataraman, M. T. Gallagher, D. Müller, J. A. West, N. F. Borrelli, D. C. Allan, K. W. Koch, Low-loss hollow-core silica/air photonic bandgap fibre. *Nature* **424**, 657–659 (2003).

8. F. Couny, F. Benabid, P. J. Roberts, P. S. Light, M. G. Raymer, Generation and photonic guidance of multi-octave optical-frequency combs. *Science* **318**, 1118–1121 (2007).

9. R. Beravat, G. K. L. Wong, M. H. Frosz, X. M. Xi, P. St., J. Russell, Twist-induced guidance in coreless photonic crystal fiber: A helical channel for light. *Sci. Adv.* **2**, e1601421 (2016).

10. J. D. Joannopoulos, S. G. Johnson, J. N. Winn, R. D. Meade, *Photonic Crystals: Molding the Flow of Light* (Princeton Univ. Press, ed. 2, 2008).

11. S. Noda, K. Kitamura, T. Okino, D. Yasuda, Y. Tanaka, Photonic-crystal surface-emitting lasers: Review and introduction of modulated-photonic crystals. *IEEE J. Sel. Top. Quantum Electron.* **23**, 4900107 (2017).

12. W. Liu, H. Ma, A. Walsh, Advance in photonic crystal solar cells. *Renew. Sustain. Energy Rev.* **116**, 109436 (2019).

13. T. Sylvestre, E. Genier, A. N. Ghosh, P. Bowen, G. Genty, J. Troles, A. Mussot, A. C. Peacock, M. Klimczak, A. M. Heidt, J. C. Travers, O. Bang, J. M. Dudley, Recent advances in supercontinuum generation in specialty optical fibers [Invited]. *J. Opt. Soc. Am. B* **38**, F90–F103 (2021).

14. V. S. Chaudhary, D. Kumar, B. P. Pandey, S. Kumar, Advances in photonic crystal fiber-based sensor for detection of physical and biochemical parameters—A review. *IEEE Sens. J.* **23**, 1012–1023 (2023).

15. T. Ozawa, H. M. Price, A. Amo, N. Goldman, M. Hafezi, L. Lu, M. C. Rechtsman, D. Schuster, J. Simon, O. Zilberberg, I. Carusotto, Topological photonics. *Rev. Mod. Phys.* **91**, 015006 (2019).

16. G.-J. Tang, X.-T. He, F.-L. Shi, J.-W. Liu, X.-D. Chen, J.-W. Dong, Topological photonic crystals: Physics, designs, and applications. *Laser Photonics Rev.* **16**, 2100300 (2022).

17. S. Vaidya, A. Ghorashi, T. Christensen, M. C. Rechtsman, W. A. Benalcazar, Topological phases of photonic crystals under crystalline symmetries. *Phys. Rev. B* **108**, 085116 (2023).

18. W. A. Benalcazar, T. Li, T. L. Hughes, Quantization of fractional corner charge in C_n-symmetric higher-order topological crystalline insulators. *Phys. Rev. B* **99**, 245151 (2019).

19. T. Li, P. Zhu, W. A. Benalcazar, T. L. Hughes, Fractional disclination charge in two-dimensional C_n-symmetric topological crystalline insulators. *Phys. Rev. B* **101**, 115115 (2020).

20. C. W. Peterson, T. Li, W. Jiang, T. L. Hughes, G. Bahl, Trapped fractional charges at bulk defects in topological insulators. *Nature* **589**, 376–380 (2021).

21. Y. Liu, S. Leung, F.-F. Li, Z.-K. Lin, X. Tao, Y. Poo, J.-H. Jiang, Bulk-disclination correspondence in topological crystalline insulators. *Nature* **589**, 381–385 (2021).

22. S. Barik, H. Miyake, W. DeGottardi, E. Waks, M. Hafezi, Two-dimensionally confined topological edge states in photonic crystals. *New J. Phys.* **18**, 113013 (2016).

23. T. Ma, G. Shvets, All-Si valley-Hall photonic topological insulator. *New J. Phys.* **18**, 025012 (2016).

24. B. Bahari, A. Ndao, F. Vallini, A. E. Amili, Y. Fainman, B. Kanté, Nonreciprocal lasing in topological cavities of arbitrary geometries. *Science* **358**, 636–640 (2017).

25. Y. Zeng, U. Chattopadhyay, B. Zhu, B. Qiang, J. Li, Y. Jin, L. Li, A. G. Davies, E. H. Linfield, B. Zhang, Y. Chong, Q. J. Wang, Electrically pumped topological laser with valley edge modes. *Nature* **578**, 246–250 (2020).

26. A. Dikopoltsev, T. H. Harder, E. Lustig, O. A. Egorov, J. Beierlein, A. Wolf, Y. Lumer, M. Emmerling, C. Schneider, S. Höfling, M. Segev, S. Klembt, Topological insulator vertical-cavity laser array. *Science* **373**, 1514–1517 (2021).

27. L. Pilozzi, D. Leykam, Z. Chen, C. Conti, Topological photonic crystal fibers and ring resonators. *Opt. Lett.* **45**, 1415–1418 (2020).

28. M. Makwana, R. Wiltshaw, S. Guenneau, R. Craster, Hybrid topological guiding mechanisms for photonic crystal fibers. *Opt. Express* **28**, 30871–30888 (2020).

29. R. Gong, M. Zhang, H. Li, Z. Lan, Topological photonic crystal fibers based on second-order corner modes. *Opt. Lett.* **46**, 3849–3852 (2021).

30. H. Huang, Z. Ning, T. Kariyado, T. Amemiya, X. Hu, Topological photonic crystal fiber with honeycomb structure. *Opt. Express* **31**, 27006–27019 (2023).

31. H. Lin, L. Lu, Dirac-vortex topological photonic crystal fibre. *Light Sci. Appl.* **9**, 202 (2020).

32. N. Roberts, G. Baardink, J. Nunn, P. J. Mosley, A. Souslov, Topological supermodes in photonic crystal fiber. *Sci. Adv.* **8**, eadd3522 (2022).

33. N. D. Mermin, The topological theory of defects in ordered media. *Rev. Mod. Phys.* **51**, 591–648 (1979).

34. Z.-K. Lin, Q. Wang, Y. Liu, H. Xue, B. Zhang, Y. Chong, J.-H. Jiang, Topological phenomena at defects in acoustic, photonic and solid-state lattices. *Nat. Rev. Phys.* **5**, 483–495 (2023).

35. M.-S. Hwang, H.-R. Kim, J. Kim, B.-J. Yang, Y. Kivshar, H.-G. Park, Vortex nanolaser based on a photonic disclination cavity. *Nat. Photon.* **18**, 286–293 (2024).

36. Z. Hu, D. Bongiovanni, Z. Wang, X. Wang, D. Song, J. Xu, R. Morandotti, H. Buljan, Z. Chen, Topological orbital angular momentum extraction and twofold protection of vortex transport. *Nat. Photon.* **19**, 162–169 (2025).

37. X. Gao, L. Yang, H. Lin, L. Zhang, J. Li, F. Bo, Z. Wang, L. Lu, Dirac-vortex topological cavities. *Nat. Nanotechnol.* **15**, 1012–1018 (2020).

38. Q. Wang, H. Xue, B. Zhang, Y. D. Chong, Observation of protected photonic edge states induced by real-space topological lattice defects. *Phys. Rev. Lett.* **124**, 243602 (2020).

39. L. Yang, G. Li, X. Gao, L. Lu, Topological-cavity surface-emitting laser. *Nat. Photon.* **16**, 279–283 (2022).

40. S. Han, Y. Chua, Y. Zeng, B. Zhu, C. Wang, B. Qiang, Y. Jin, Q. Wang, L. Li, A. G. Davies, E. H. Linfield, Y. Chong, B. Zhang, Q. J. Wang, Photonic Majorana quantum cascade laser with polarization-winding emission. *Nat. Commun.* **14**, 707 (2023).

41. T. A. Loring, *K*-theory and pseudospectra for topological insulators. *Ann. Phys.* **356**, 383–416 (2015).

42. A. Cerjan, T. A. Loring, An operator-based approach to topological photonics. *Nanophotonics* **11**, 4765–4780 (2022).

43. A. Cerjan, T. A. Loring, F. Vides, Quadratic pseudospectrum for identifying localized states. *J. Math. Phys.* **64**, 023501 (2023).

44. A. Cerjan, T. A. Loring, H. Schulz-Baldes, Local markers for crystalline topology. *Phys. Rev. Lett.* **132**, 073803 (2024).

45. A. Cerjan, T. A. Loring, Classifying photonic topology using the spectral localizer and numerical *K*-theory. *APL Photonics* **9**, 111102 (2024).

46. S. Wong, T. A. Loring, A. Cerjan, Classifying topology in photonic crystal slabs with radiative environments. *npj Nanophoton.* **1**, 19 (2024).

47. J. J. Garcia, A. Cerjan, T. A. Loring, Clifford and quadratic composite operators with applications to non-Hermitian physics. arXiv:2410.03880v1 [math-ph] (2024).

48. J. Noh, T. Schuster, T. Iadecola, S. Huang, M. Wang, K. P. Chen, C. Chamon, M. C. Rechtsman, Braiding photonic topological zero modes. *Nat. Phys.* **16**, 989–993 (2020).

49. Q. Wang, Y. Ge, H. Sun, H. Xue, D. Jia, Y. Guan, S. Yuan, B. Zhang, Y. D. Chong, Vortex states in an acoustic Weyl crystal with a topological lattice defect. *Nat. Commun.* **12**, 3654 (2021).

50. L. H. Wu, X. Hu, Scheme for achieving a topological photonic crystal by using dielectric material. *Phys. Rev. Lett.* **114**, 223901 (2015).

51. P. M. Chaikin, T. C. Lubensky, *Principles of Condensed Matter Physics* (Cambridge Univ., 1995).

52. R. G. H. van Uden, R. A. Correa, E. A. Lopez, F. M. Huijskens, C. Xia, G. Li, A. Schülzgen, H. de Waardt, A. M. J. Koonen, C. M. Okonkwo, Ultra-high-density spatial division multiplexing with a few-mode multicore fibre. *Nat. Photon.* **8**, 865–870 (2014).

53. Z.-G. Chen, R.-Y. Zhang, C. T. Chan, G. Ma, Classical non-Abelian braiding of acoustic modes. *Nat. Phys.* **18**, 179–184 (2022).

54. N. Roberts, B. Salter, J. Binysh, P. J. Mosley, A. Souslov, Twisted fibre: A photonic topological insulator. arXiv:2411.13064 [physics.optics] (2024).

55. A. P. Thompson, H. M. Aktulga, R. Berger, D. S. Bolintineanu, W. M. Brown, P. S. Crozier, P. J. in ‘t Veld, A. Kohlmeyer, S. G. Moore, T. D. Nguyenf, R. Shan, M. J. Stevens, J. Tranchida, C. Trott, S. J. Plimpton, LAMMPS—A flexible simulation tool for particle-based materials modeling at the atomic, meso, and continuum scales. *Comput. Phys. Commun.* **271**, 108171 (2022).

56. J.-K. Yang, C. Schreck, H. Noh, S.-F. Liew, M. I. Guy, C. S. O'Hern, H. Cao, Photonic-band-gap effects in two-dimensional polycrystalline and amorphous structures. *Phys. Rev. A* **82**, 053838 (2010).

57. H.-X. Wang, G.-Y. Guo, J.-H. Jiang, Band topology in classical waves: Wilson-loop approach to topological numbers and fragile topology. *New J. Phys.* **21**, 093029 (2019).

58. M. Blanco de Paz, C. Devescovi, G. Giedke, J. J. Saenz, M. G. Vergniory, B. Bradlyn, D. Bercioux, A. García-Etxarri, Tutorial: Computing topological invariants in 2D photonic crystals. *Adv. Quantum Technol.* **3**, 1900117 (2020).

59. T. Neupert, F. Schindler, *Topological Matter* (Springer International Publishing, 2018), pp. 31–61.

60. D. Vanderbilt, *Berry Phases in Electronic Structure Theory: Electric Polarization, Orbital Magnetization and Topological Insulators* (Cambridge Univ. Press, 2018).

61. A. Cerjan, L. Koekenbier, H. Schulz-Baldes, Spectral localizer for line-gapped non-Hermitian systems. *J. Math. Phys.* **64**, 082102 (2023).

62. F. Poletti, Nested antiresonant nodeless hollow core fiber. *Opt. Express* **22**, 23807–23828 (2014).
